# Equivalent impacts of logging and beaver activities on aboveground carbon stock loss in the southernmost forest on Earth

**DOI:** 10.1038/s41598-023-45657-4

**Published:** 2023-10-26

**Authors:** Alejandro Miranda, Jorge Hoyos-Santillan, Antonio Lara, Rayén Mentler, Alejandro Huertas-Herrera, Mónica D. R. Toro-Manríquez, Armando Sepulveda-Jauregui

**Affiliations:** 1 Center for Climate and Resilience Research (CR), Santiago, Chile; 2 Laboratorio de Ecología del Paisaje y Conservación, Departamento de Ciencias Forestales, Universidad de La Frontera, Temuco, Chile; 3 School of Biosciences, University of Nottingham, Sutton, Bonington UK; 4 Environmental Biogeochemistry Laboratory, GAIA Antarctica Research Centre (CIGA-UMAG), University of Magallanes, Punta Arenas, Chile; 5 Facultad de Ciencias Forestales y Recursos Naturales, Instituto de Conservación, Biodiversidad y Territorio, Universidad Austral de Chile, Valdivia, Chile; 6 Fundación Centro de los Bosques Nativos FORECOS, Valdivia, Chile; 7 Grupo Ecología Forestal, Centro de Investigación en Ecosistemas de la Patagonia (CIEP), Coyhaique, Chile

**Keywords:** Ecosystem services, Environmental impact, Climate-change mitigation, Climate-change policy, Environmental impact

## Abstract

The conservation of forest landscapes is crucial for global climate strategies, and the forest in Tierra del Fuego, located in Patagonia, represents the southernmost example on Earth. These ecosystems are critical for Chile’s roadmap toward carbon neutrality. Unfortunately, these ecosystems have been impacted by logging and beaver activities. Currently, the precise contribution of each driver to forest cover and carbon stock loss remains insufficiently quantified, impeding effective policymaking and the implementation of strategies to safeguard and enhance carbon stocks in these ecosystems. In this study, we conducted an assessment of forest carbon stock loss resulting from both logging and beaver activities in Chilean Tierra del Fuego from 1986 to 2019. While beavers have received significant attention for their substantial contribution to forest cover loss (56.1% forest cover, ≈ 1.4 MtC), our findings suggest that logging has nearly equally contributed to carbon stock depletion (43.8% forest cover, ≈ 1.2 MtC). Consequently, the prevailing focus on beavers has obscured the ongoing logging-induced carbon stock loss. The implications of our study highlight the urgency for comprehensive consideration of both drivers in Chile’s climate strategy to fulfill the country’s mitigation commitments.

## Introduction

Native forest is the largest repository of terrestrial biodiversity on Earth, and its protection, management, and restoration are among the most effective Nature-based Solutions (NbS) alternatives to mitigate climate change^1^. However, the carbon stock and sink capacity of native forests are threatened by climate stress, biotic agents, and human activities^2,3^. At present, approximately 35% of the Earth’s cover of pre-industrial native forest has been lost^4^ and less than 20% remains as Intact Forest Landscape^2^.

Industrial timber extraction, agricultural expansion, wildfires, land-use change for infrastructure development, livestock grazing, mining, and oil and gas projects are the primary threats driving forest loss worldwide^5,6^. Evaluating the net contribution of each driver to the loss of forest is among the first steps necessary for developing and implementing effective policies focused on preserving these ecosystems^6,7^. The largest forest loss currently occurs in tropical South America, where industrial timber extraction and agricultural expansion are the main causes of forest loss^8^. In addition, other drivers, such as invasive species introduced by humans, have played a relevant role in forest loss in the remotest areas of South America, such as southern Patagonia^9^.

Southern Patagonia (> 50° S) is considered among the least disturbed landscapes globally^2^ and, comprising ≈ 20% of Chile’s native forest^10^, is a major component of Chile’s climate strategy toward carbon neutrality^11^. For instance, the country’s Nationally Determined Contributions (NDC) include the sustainable management of 200,000 ha of native forest, an additional 100,000 ha of industrial plantations, the afforestation of 100,000 ha (70% native forest and 30% non-native species), the reduction of forest degradation and deforestation associated with forestry, and the development of a National Plan for the Restoration of Landscapes comprising 1 million ha^12^. However, in recent decades, the impact of introduced beavers has been recognized as a significant driver of forest loss in the Tierra del Fuego archipelago (TdF)^13–15^. Thus, beavers are currently threatening the carbon stock and sink capacity of the southernmost native forest on the planet, which is also among the most vulnerable and irrecoverable biomass carbon stocks on Earth^16,17^.

Beavers were introduced into the main island of Tierra del Fuego in 1946 by the Argentinian government; since then, their population increased from 50 to over 100,000 individuals^18,19^. At present, beavers have colonized 98% of the watersheds in TdF^20^ and have built more than 200,000 dams (105,252 and 100,951 dams in the Chilean and Argentinian parts of TdF, respectively)^15^. This colinization has negatively impacted the native forest cover and its aboveground carbon (AGC) stock because beavers use trees for foraging and as material to build dams. In areas impacted by beavers, trees biomass and carbon pools are rearranged. As trees die, the amount of alive trees decreases significantly, whereas standing dead biomass and lying dead biomass increase. This dead biomass slowly decomposes over time, releasing carbon into the atmosphere^21^.

But beavers are not the only threat to TdF forest. Since the nineteenth century, TdF has been subjected to sheep grazing, logging, and mining activities^22^. Nowadays, logging for commercial purposes remains an industrial economic activity in TdF^23^. Therefore, it has become necessary to quantitatively assess the impact that different drivers exert on different ecosystems' carbon reservoir and sink capacity as NbS emerge as a central components of carbon neutrality roadmaps worldwide^24^. In this study, we assess the impact of logging and beavers on the cover and AGC stock of native forests in Chilean Tierra del Fuego, as these two are the main drivers of forest disturbance in the study area. Our assessment is based on remote sensing data, machine learning algorithms, and carbon stock data available in the literature. Specifically, we aimed to (i) evaluate the relative contribution of logging and beavers to the loss of native forest cover and (ii) estimate how these drivers have impacted the AGC stocks of native forests, both between 1986 and 2019.

## Results

### Forest cover loss

The global accuracy assessment of image classification showed 96%, 95%, and 93% accuracy rates for the years 1986, 2002, and 2019, respectively. Based on the confusion matrix, our predictive model for forest loss caused by logging and beavers had an overall accuracy of 95.2%, consistent with an AUC result of 95.4% (Kappa = 88.2%). The producer accuracy for beaver was the most accurate, while the omission error for logging was the biggest error, underestimating this class (Table 1). Commission errors were similar between both classes.

**Table 1:** Accuracy assessment for forest loss drivers model.

Driver	User accuracy	Commission error	Producer accuracy	Omission error
Logging	0.96	0.04	0.88	0.12
Beaver	0.95	0.05	0.98	0.016

**Figure 1:**
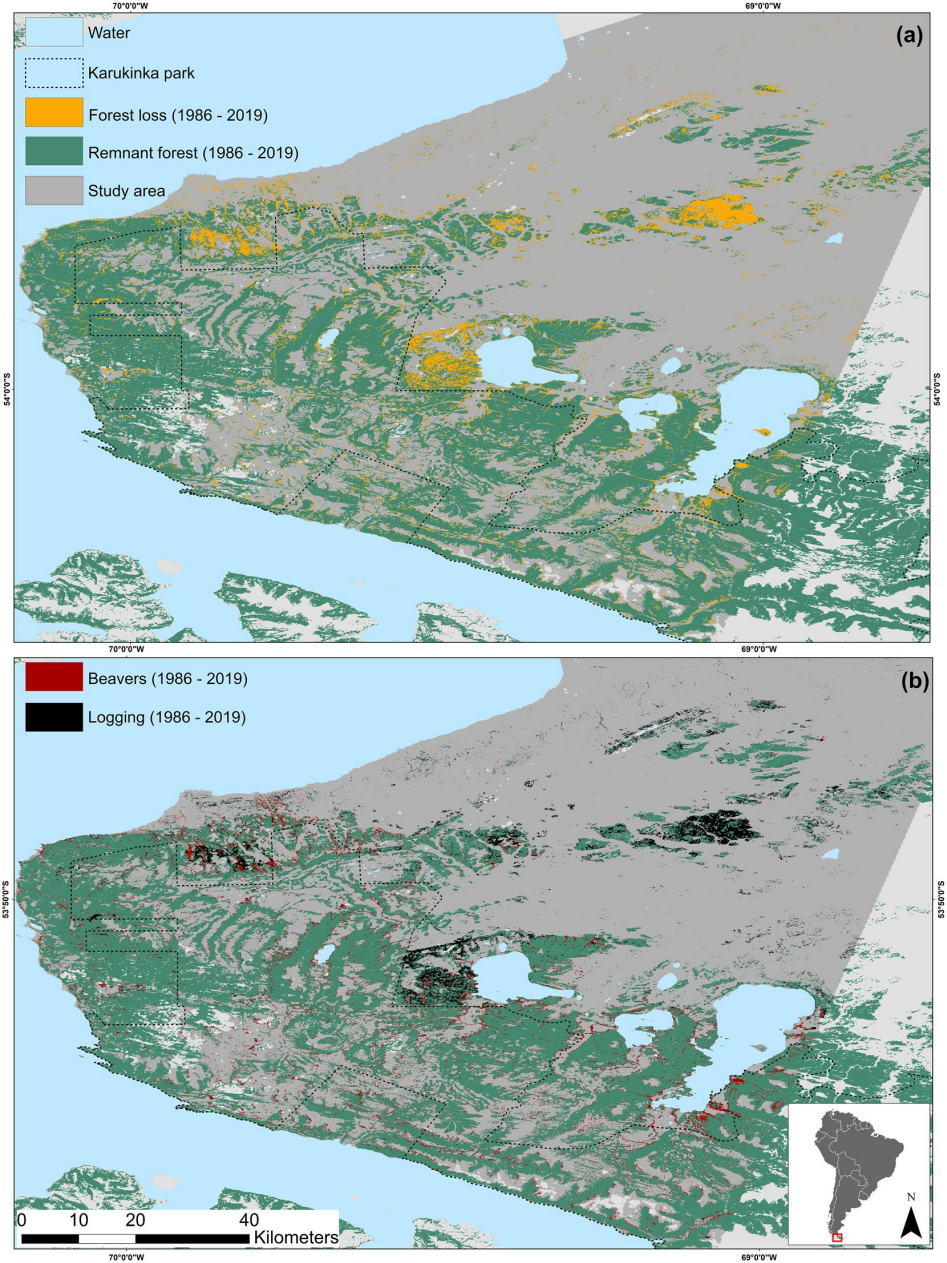
(**a**) Forest loss in the study area and (**b**) predicted drivers of forest loss by logging and beaver activities in Tierra del Fuego. Classification models were developed using the Google Earth Engine platform (https://earthengine.google.com/) and maps were generated using QGIS Geographic Information System 3.18.3 (Open Source Geospatial Foundation Project. http://qgis.org).

According to standard land cover change analysis, we estimated a total forest cover loss of 15.5% in the study area between 1986 and 2019 (Fig. 1, Table 2). Beavers were responsible for a larger proportion of forest cover loss than logging (56.1% vs. 43.8%). (Fig. 1, Table 2). The spatial distribution of forest loss due to each driver was influenced by beavers’ dam density (39.7%), distance to roads (15.5%), and latitude (10.9%). Beavers’ impact was particularly significant in lowlands near water streams, lakes, and peatlands, while logging impact was clustered near roads (mean distance < 2.5 km). Logging impact was more pronounced in the northern portion of the study area, while beavers’ impact was more significant in the southern, remote areas (Fig. 1).

**Table 2:** Loss of forest cover and aboveground carbon (AGC) in Tierra del Fuego.

Period	Driver	Forest cover loss (ha)^a^	Forest AGC stock loss (MtC)^b,c^
1986–2002	Logging	7299	0.60 ± 0.06
2002–2019	Logging	7614	0.61 ± 0.07
1986–2002	Beaver	10,624	0.80 ± 0.14
2002–2019	Beaver	8458	0.64 ± 0.11
	Total	33,995	2.66 ± 0.17

^a^Study area: 0.7 million ha; native forest cover within the study area: 220,000 ha.

^b^Mean ± SE.

^c^Total pre-disturbance AGC stock in the impacted area in 1986: 5.91 MtC.

**Figure 2:**
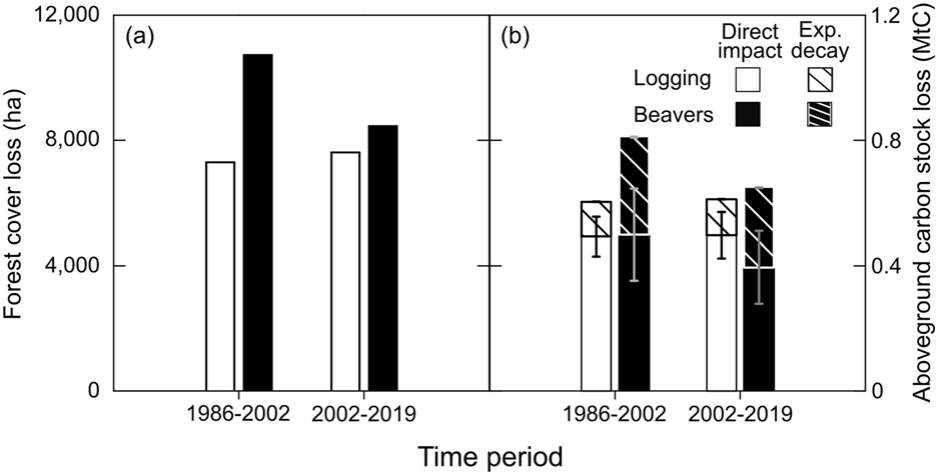
(**a**) Forest loss (ha) due to logging (white bars) and beaver activities (black bars) between 1986–2002 and 2002–2019; (**b**) aboveground carbon (AGC) stock loss (mean ± SE) due to the direct impact of logging and beaver activities (solid bars) and due to exponential decay of aboveground dead biomass (striped bars), between 1986–2002 and 2002–2019.

Temporal analysis showed that 53% of the forest cover loss occurred from 1986 to 2002, while 47% occurred from 2002 to 2019 (Table 2, Fig. 2a). Beavers were the primary contributor to forest cover loss from 1986 to 2019, but their impact decreased by 20.39% from 1986–2002 to 2002–2019 (Table 2). Conversely, logging impact increased marginally by 4.32% over the same period (Table 2).

### Potential carbon stock loss

**Figure 3:**
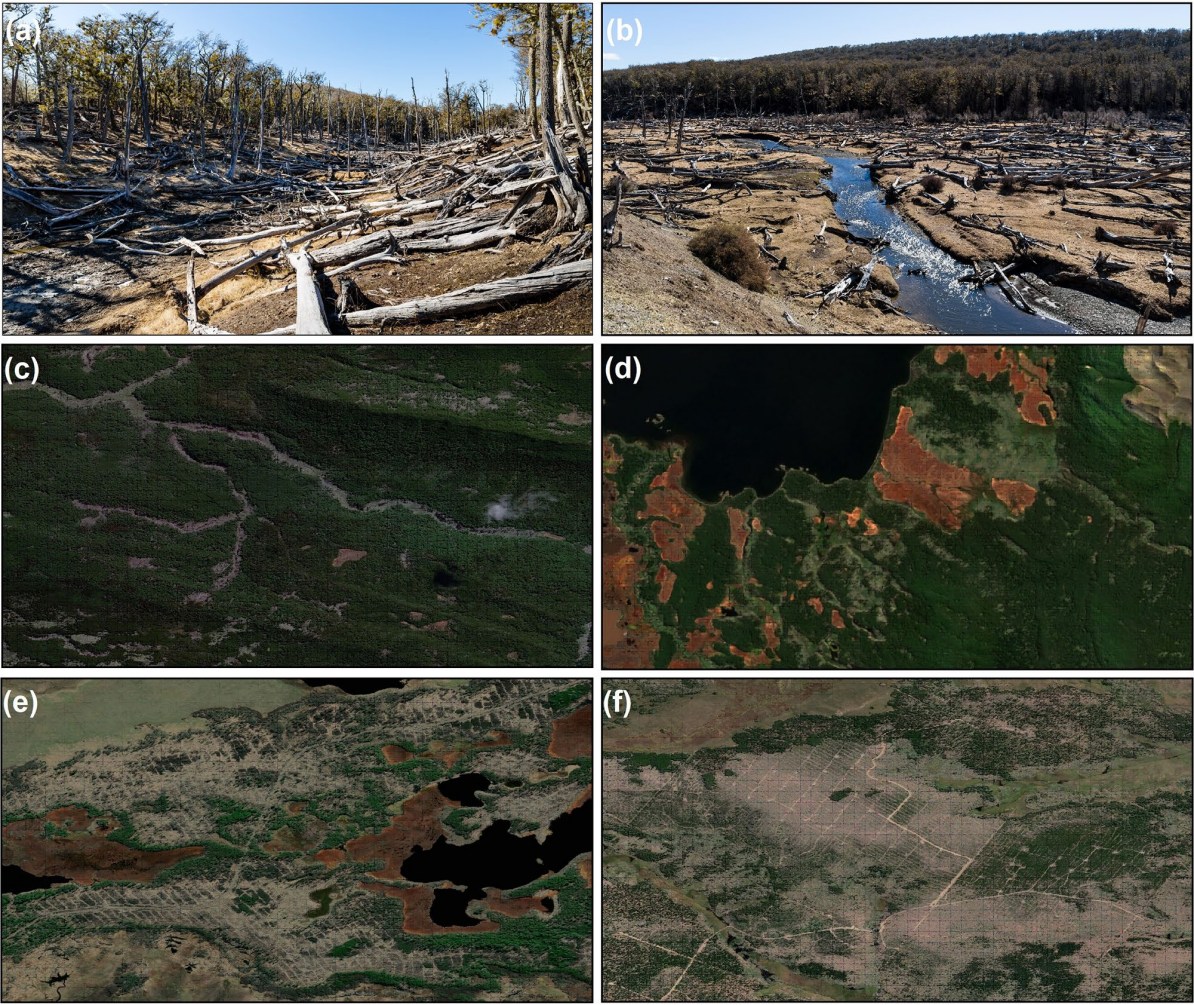
Examples of forest loss due to logging and beaver activities: (**a,b**) streams affected by beaver activities with aboveground dead biomass; (**c,d**) aerial images depicting beaver impact adjacent to major water streams; and (**e,f**) logging plots. Satellite imagery was obtained from Google Earth Pro 7.3 (https://earth.google.com/).

In 1986, the forest within the study area had an AGC stock of approximately 38.11 MtC; but logging and beaver activity only affected a forest area holding an undisturbed AGC stock of 5.91 MtC. Based on the comparison between undisturbed and disturbed AGC stocks of forests affected by logging and beaver activity, we estimated that logging and beavers contributed to a carbon loss of 1.21 ± 0.14 MtC (i.e., 4.45 ± 0.51 MtCO_2_) and 1.44 ± 0.21 MtC (i.e., 5.31 ± 0.90 MtCO_2_), respectively (Table 2, Fig. 2b). The mechanism of carbon loss was different between the two drivers. Beavers redistributed the forest carbon pools by decreasing the aboveground live biomass and increasing the aboveground dead biomass (Figs. 2b, 3a–d). Logging, on the other hand, represented a direct carbon extraction from the ecosystem due to harvesting practices (Figs. 2b, 3e,f). Native forests impacted by logging lost 47% of their AGC stock, whereas native forests impacted by beaver activity lost 44% of their original AGC stock.

## Discussion

Despite being the southernmost Intact Forest Landscape on the planet^2,25^, intensive forest cover loss related to beavers and logging has occurred for at least four decades in Tierra del Fuego (TdF). Our study highlights the significant impact of beavers on forest cover loss, with a 56% loss associated with beaver activity and a 44% loss associated with logging in Chilean TdF. However, in contrast to the general idea that beavers are by far the main threat to forest cover loss^9,14,15^, our results suggest that beavers and logging had a similar impact on carbon stock loss between 1986 and 2019. While logging removes most of the aboveground carbon (AGC) stock, a substantial amount of the AGC stock remains as deadwood in areas impacted by beavers, where it gradually decomposes over time^26^ (Fig. 2b).

Our estimation considers a conservative value of carbon stock loss due to logging, based on the extraction of 37% of the forest basal area. This is based on shelterwood cuts, which are the most commonly used harvesting method in TdF, with harvesting intensity varying between 30 and 60%^27,28^. However, during our analysis, we observed higher forest cover loss due to intensive logging or indirect damage possibly caused by blowdown events after harvesting (Fig. 3)^28^.

On the other hand, our analysis of carbon stock loss does not consider the potential natural regeneration of forests. While natural regeneration can contribute to restoring forest structure, increasing biodiversity, and sequestering carbon^29–31^, its occurrence varies depending on the driver of forest disturbance^32^. For example, flooding associated with beaver dams affects forest structure, biomass allocation, biodiversity, and carbon fluxes^14,33^. Consequently, as beavers develop new stable ecosystems with different soil moisture conditions, light availability, and plant communities, these conditions limit the natural and assisted regeneration of *N. pumilio* forests^13^. Similarly, logging affects ecosystem processes and biodiversity, increasing the susceptibility of the remaining forest to be impacted by blowdown events, biological invasions, freezing, drought, or browsing^34,35^. Some of these impacts are long-lasting and must be considered when designing restoration initiatives focused on carbon sequestration. The recovery of carbon stock in Patagonian forests is a lengthy process that can span centuries. For instance, *N. pumilio* individual trees may require between 110 and 170 years to reach a diameter at breast height (d.b.h.) of 40 cm^36^, and it can take over 230 years to accumulate more than 400 t ha^-1^ of biomass, depending on site quality^37^. Furthermore, naturally regenerated *N. pumilio* stands in Tierra del Fuego are impacted by herbivory^35^, leading to a reduction in biomass accumulation. In a 50 years old *N. pumilio* secondary forest impacted by guanacos (*Lama guanicoe*), tree heights ranged from 0.6 to 1.6 m, whereas undisturbed *N. pumilio* stands reached heights between 10 and 14 m^38^. Similarly, as much as 81.5% of *N. pumilio* seedlings growing in forests transformed into meadows by beaver activity displayed signs of browsing by guanacos^34^. Although our study suggests a decrease in the impact of beavers over time, this decline may be attributed to joint efforts in both Chile and Argentina aimed at controlling beaver populations and restoring impacted ecosystems^39^. Between 2005 and 2006, Chile captured 11,700 beavers, and in 2008, a binational agreement was signed between Chile and Argentina to control the beaver population and restore the impacted ecosystems. In this context, Chile co-sponsored a US$ 7.8 million project with the Global Environmental Facility to control the beaver population in Chilean Patagonia^40^. Moreover, it is possible that the saturation of colonizable areas has led to the colonization of the steppe and the dispersal of beavers to neighboring islands and the continent^13,41^.

Previous analyses of the effect of beaver invasion estimated a forest cover loss of 31,476 ha in the Argentine portion of TdF caused by direct cutting of riparian forest and tree removal or flooding near beaver ponds^14^. In addition, it has been estimated that logging activities have caused the loss of 31,000 ha of forest in the same area since European colonization, and other human impacts have led to the additional loss of 21,000 ha of forest until 1995^42^. Our results show a similar pattern, but a lower magnitude for the Chilean part of TdF, with 19,184 ha of forest loss associated with beaver activity and 14,913 ha due to logging. Together, forest loss in TdF for both Argentina and Chile reveals that human activity must not be underestimated in conservation or restoration programs considering the overall anthropogenic impact in Patagonia.

Forest perturbation could also potentially affect other carbon reservoirs in TdF. In TdF, forest is adjacent to peatlands (Fig. 3), which are the largest terrestrial carbon reservoir in Chile. Peatlands hold approximately 5 times more carbon than all the aboveground biomass in all the forests and plantations in the country^11^. At present, however, it is not possible to ascertain whether forest cover loss has had a negative impact on the carbon stock and carbon sink capacity of Patagonian peatlands. The impact of beavers on the overall carbon balance of Patagonian peatlands also remains uncertain. While beavers can promote carbon sequestration in semiarid ecosystems in the Northern Hemisphere by expanding riparian meadows^43^, their impact in Patagonia is distinct. Here, beavers create channels that partially drain the peatlands^44^, resulting in a lowering the water table and, consequently, an alteration of the carbon dynamics within the ecosystem. Future research should explore the relationship between Patagonian forests and the stability of carbon stocks in distinct subantarctic ecosystems.

In Chile, the current climate strategy to achieve carbon neutrality heavily relies on native forests, which constitute the only net carbon sink in the national greenhouse gas inventory^11^. Thus, to fulfill its NDC, Chile will have to protect its forest and work toward increasing its carbon sink capacity. This gives further importance to Patagonian forest which comprises more than 70% of Chile’s native forest and represents the largest Intanct Forest Landscape in southern South America^2^. Gaining knowledge on the drivers and mechanisms of natural forest loss in Patagonia will help develop adequate policies and programs focused on the conservation, restoration, and management of these unique ecosystems.

## Materials and methods

### Study area

Our study area is located in Patagonia, encompassing 0.7 million hectares within the Chilean portion of the Tierra del Fuego (TdF) archipelago, the southernmost region of South America before Antarctica (Fig. 1). The archipelago is considered one of the world's intact global ecosystems^2,4^.

The forest in the study area is dominated by *Nothofagus pumilio* (lenga), which comprises 70% of the total forest area^10^. The remaining forest is made up of *N. betuloides* (coigüe de Magallanes) and *N. antarctica* (ñirre). About 77% of the forest is considered old-growth^10^. *N. pumilio* is the most affected tree species in TdF due to beaver activity, both directly (*e.g*., building dams and foraging) and indirectly (long-term flooding)^45,46^.

### Land cover change analysis

We analyzed forest cover loss between 1986–2002 and 2002–2019 using Landsat satellite images with 30 m spatial resolution in Google Earth Engine (GEE)^47^. Images were classified using the following categories: forest, peatlands, barelands, shrublands, grasslands-pasturelands, and water bodies. As we use pixel-based sampling and classification, we exclusively utilize pixels that are entirely covered by the specific land cover class. The land cover classification was conducted using the Random Forest algorithm with a 50 decision tree parameter. Training and testing points were obtained using high-resolution 2019 Sentinel-2 and Google Earth images. Since high-resolution images are only available for the most recent images, the sampling was mainly performed in areas without land cover change considered as stable samples^48^. The classifier was trained and tested with 70% and 30% of the sample, respectively (N_1986_ = 1029; N_2002_ = 1800; N_2019_ = 1381). We used testing points and utilized the standard contingency table to assess the accuracy of satellite image classification for each year.

### Identification and classification of forest loss drivers

Various drivers of forest cover loss have been documented in Tierra del Fuego, including logging, beaver activity, wildfires, blowdowns resulting from windstorms, and even earthquakes leading to forest gap formation^49^. These drivers operate on differential spatial and temporal scales^14,23,49^. We excluded wildfires from this analysis due to the limited burned forest area, approximately 8 hectares, reported by the Chilean Forest Service (CONAF) within the study area and during the study timeframe^50^. Blowdown events in Tierra del Fuego, as a consequence of storms, have occurred regularly but on a long-term scale. Rebertus et al.^35^ identified two major storms in the last century, one in 1972 and another in 1924, impacting a single forest patch of approximately 100 ha. They estimated that blowdown events occurred at an approximate frequency of every 20 to 30 years. Additionally, Rebertus and Veblen^49^ described gap formation in the forest as highly episodic, with gaps predominantly smaller than 0.02 ha. They also observed larger individual gaps ranging from 0.12 to 0.17 ha across all sites. The reduction in forest density due to logging or beaver activity leaves the remaining trees exposed to blowdown events. We considered blowdown events located within logging and beaver activity impacted areas as a direct effect of these drivers.

We used 250 × 250 m sample grids over the classified images to differentiate forest cover loss drivers. We randomly selected 10% of the 12,285 grid cells with forest loss > 1 ha occurring between 1986 and 2019. Three independent observers visually interpreted each grid to determine whether it was lost due to logging or beaver activity. To discriminate between drivers, we used Augmented Visual Interpretation, which involves multiple sources of information for land cover change analysis^51^. Observers’ classification was conducted using a cloud-free mosaic Sentinel-2 satellite imagery in GEE from the 2019 to 2020 time series. We complemented the visual interpretation from Sentinel-2 imagery with higher-resolution aerial imagery from Bing aerial maps (Microsoft, USA) and ESRI Satellite imagery available in QGIS. A similar approach has been used in TdF to map beaver pounds^20^ and analyze the invasion status of beavers and the environmental variability related to beaver colonization^14,15^. We also used contextual information to improve driver’s classification, including topography, density and location of beavers’ ponds, and road distribution. The three independent observers agreed in 56% of a classified grid cell, 379 due to beaver activity and 266 due to logging used to fit a drivers of forest loss prediction model.

The forest cover loss drivers classification prediction model had logging and beaver activity as response variables and was fitted using the Boosted Regression Trees method (BRT)^7^. Initially, we used (i) climate, (ii) topography and geography, (iii) indicators of human and beaver activity, and (iv) land cover characteristics relevant for logging and beaver activity as predictor variables groups (Supporting Information 1). To consider the large number of predictors and balance the learning rate and the decision tree complexity parameters, we fitted models with > 1000 trees^52^. The model parameters were defined as: tree complexity = 5, learning rate = 0.001, bag fraction = 0.7, and maximal tress = 20,000. We retained those variables with relative importance > 2%. Model classification accuracy assessment was evaluated using the confusion matrix and Area Under the Curve (AUC) approaches.

The model was trained using the 645 grid cells on which the independent observers coincided on their classification. The process was computed using the R ‘*dismo’* package^53^. Finally, the model was predicted in the set of 57,726 sample grids with 101 iterations and, through a simple majority assessment, the most likely cause was assigned for each sample grid.

### Carbon stock loss

For this study, AGC stock comprised only the forest’s three major carbon pools for aboveground biomass (AGB): live tree biomass, standing deadwood, and lying deadwood. We used *N. pumilio* as model for our AGB and AGC estimations as it was the dominant species in the study area, it is the most impacted by commercial logging, and beavers prefer it. Altitudinal heterogeneity of AGB in TdF forest, decreasing with elevation, was incorporated in the analysis by considering four elevation ranges above mean sea level (amsl) with distinct average AGB: 0–220 m (492.5 t_biomass_ ha^−1^), 221–440 m (349.8 t_biomass_ ha^−1^), 441–540 m (132.5 t_biomass_ ha^−1^), and > 541 m (30.4 t_biomass_ ha^−1^)^54^. We estimated the AGC using the AGB for each carbon pool within each altitude range in conjunction with a *N. pumilio* biomass/carbon content factor of 1 kg AGB = 0.457 kg C^54^. Ratios for the distribution of the three AGC pools for disturbed and undisturbed *N. pumilio* forests were estimated according to Harris et al. and applied to the altitudinal ranges (Supporting information 2.1). The AGC loss in disturbed forests by logging or beaver activity included: (i) the direct carbon loss associated with the initial disturbance and (ii) the carbon loss due to the exponential decay of wood within the impacted area (Supporting Information 2.2). Carbon emissions (tC ha^−1^) associated with the decay of dead biomass were estimated using exponential decay models for TdF *N. pumilio* biomass^21^ (Supporting Information 2.3). Between 1986–2002 and 2002–2019, we estimated an average constant annual forest cover loss for each driver. For this purpose, the total forest cover loss due to each driver in each period was divided over the number of years during which it occurred. We used propagation of error (SDy) to take into account the effect of the AGC stock uncertainty, using the standard deviation (SD) associated with the AGC stock calculated by Harris et al.^26^ in disturbed and undisturbed *N. pumilio* forests.

### Supplementary Information

Supplementary Information 1.

Supplementary Information 2.

## Data Availability

The datasets used and/or analysed during the current study available from the corresponding author on reasonable request.

## References

[1] Bastin J-F (2019). The global tree restoration potential. Science.

[2] Potapov P (2017). The last frontiers of wilderness: Tracking loss of intact forest landscapes from 2000 to 2013. Sci. Adv..

[3] Fei S, Morin RS, Oswalt CM, Liebhold AM (2019). Biomass losses resulting from insect and disease invasions in US forests. Proc. Natl. Acad. Sci..

[4] Watson JEM (2018). The exceptional value of intact forest ecosystems. Nat. Ecol. Evol..

[5] Fa JE (2020). Importance of Indigenous Peoples’ lands for the conservation of intact forest landscapes. Front. Ecol. Environ..

[6] Grantham HS (2021). The emerging threat of extractives sector to intact forest landscapes. Front. For. Glob. Change.

[7] Altamirano A (2020). Natural forests loss and tree plantations: Large-scale tree cover loss differentiation in a threatened biodiversity hotspot. Environ. Res. Lett..

[8] Curtis PG, Slay CM, Harris NL, Tyukavina A, Hansen MC (2018). Classifying drivers of global forest loss. Science.

[9] Choi C (2008). Tierra del Fuego: The beavers must die. Nature.

[10] CONAF. *Catastro y Evaluación de los Recursos Vegetacionales Nativos de Chile*. https://www.conaf.cl/nuestros-bosques/bosques-en-chile/catastro-vegetacional/ (2021).

[11] Hoyos-Santillan J (2021). Diversifying Chile’s climate action away from industrial plantations. Environ. Sci. Policy.

[12] Gobierno de Chile. *Chile’s Nationally Determined Contribution (NDC)—Update 2020*. https://unfccc.int/sites/default/files/NDC/2022-06/Chile%27s_NDC_2020_english.pdf (2020).

[13] Anderson CB, Griffith CR, Rosemond AD, Rozzi R, Dollenz O (2006). The effects of invasive North American beavers on riparian plant communities in Cape Horn, Chile. Biol. Conserv..

[14] Henn JJ, Anderson CB, Martínez Pastur G (2016). Landscape-level impact and habitat factors associated with invasive beaver distribution in Tierra del Fuego. Biol. Invas..

[15] Huertas Herrera A, Lencinas MV, Toro Manríquez M, Miller JA, Martínez Pastur G (2020). Mapping the status of the North American beaver invasion in the Tierra del Fuego archipelago. PLoS ONE.

[16] Goldstein A (2020). Protecting irrecoverable carbon in Earth’s ecosystems. Nat. Clim. Change.

[17] Noon ML (2021). Mapping the irrecoverable carbon in Earth’s ecosystems. Nat. Sustain..

[18] Jusim P, Goijman AP, Escobar J, Carranza ML, Schiavini A (2020). First test for eradication of beavers (*Castor canadensis*) in Tierra del Fuego, Argentina. Biol. Invas..

[19] Skewes O (2006). Abundance and distribution of American beaver, *Castor canadensis* (Kuhl 1820), in Tierra del Fuego and Navarino islands, Chile. Eur. J. Wildl. Res..

[20] Eljall A, Dieguez H, Menvielle MF, Hodara K (2019). Distribución y patrones espaciales del impacto de un ingeniero de los ecosistemas exótico e invasor, *Castor canadensis*, en Tierra del Fuego, Argentina. Ecol. Austral.

[21] Frangi JL, Richter LL, Barrera MD, Aloggia M (1997). Decomposition of *Nothofagus* fallen woody debris in forests of Tierra del Fuego, Argentina. Can. J. For. Res..

[22] Martinic M (1973). Panorama de la colonización en Tierra del Fuego entre 1881–1900. An. Inst. Patagon..

[23] Gea-Izquierdo G, Pastur GM, Cellini JM, Lencinas MV (2004). Forty years of silvicultural management in southern *Nothofagus pumilio* primary forests. For. Ecol. Manag..

[24] Girardin CAJ (2021). Nature-based solutions can help cool the planet—If we act now. Nature.

[25] Buma B, Holz A, Diaz I, Rozzi R (2021). The world’s southernmost tree and the climate and windscapes of the southernmost forests. Ecography.

[26] Harris NL, Pearson TRH, Brown S (2008). Assessing the Potential for Generating Carbon Offsets in Wildlife Conservation Society’s Karukinka reserve.

[27] Martínez Pastur GJ (2019). Knowledge arising from long-term research of variable retention harvesting in Tierra del Fuego: Where do we go from here?. Ecol. Process..

[28] Paredes D (2020). Influencia del paisaje en las cortas de protección en bosques de *Nothofagus pumilio* en Tierra del Fuego, Argentina: Cambios en la estructura forestal y respuesta de la regeneración. Bosque Valdivia.

[29] Chaves JE (2023). Carbon pool dynamics after variable retention harvesting in *Nothofagus pumilio* forests of Tierra del Fuego. Ecol. Process..

[30] Toro-Manríquez MDR, Huertas Herrera A, Soler RM, Lencinas MV, Martínez Pastur GJ (2023). Combined effects of tree canopy composition, landscape location, and growing season on *Nothofagus* forest seeding patterns in Southern Patagonia. For. Ecol. Manag..

[31] Toro-Manríquez MDR (2022). Inferring population dynamic trends of *Nothofagus pumilio* and *N. betuloides* in coastal and mountain forests of Tierra del Fuego: Contrasting from flowering to seedling survival through several reproductive cycles. Trees.

[32] Toro Manríquez MDR (2019). Suitable conditions for natural regeneration in variable retention harvesting of southern Patagonian *Nothofagus pumilio* forests. Ecol. Process..

[33] Papier CM, Poulos HM, Kusch A (2019). Invasive species and carbon flux: The case of invasive beavers (*Castor canadensis*) in riparian *Nothofagus forests* of Tierra del Fuego, Chile. Clim. Change.

[34] Manríquez, M. T. *Regeneración de Lenga en Micrositios de sectores perturbados y no perturbados por efectos del Castor canadensis Kuhl en Tierra del Fuego*. 10.13140/RG.2.1.1700.3042 (2014).

[35] Rebertus AJ, Kitzberger T, Veblen TT, Roovers LM (1997). Blowdown history and landscape patterns in the andes of Tierra del Fuego, Argentina. Ecology.

[36] Martinez Pastur G (2004). Turno de corta y posibilidad de los bosques de lenga (*Nothofagus pumilio*) en Tierra del Fuego (Argentina). Bosque Valdivia.

[37] Frangi J, Frangi J (2004). Ecología de los Bosques de la Tierra del Fuego. Ecología y manejo de los Bosques de Argentina.

[38] Martinez-Pastur G, Peri PL, Fernandez MC, Staffieri G, Rodriguez D (1999). Desarrollo de la regeneración a lo largo del ciclo del manejo forestal de un bosque de *Nothofagus pumilio*: 2. Incidencia del ramoneo de *Lama guanicoe*. Bosque.

[39] González-Calderón A, Escobar J, Deferrari G, Schiavini A (2023). Demographic plasticity in an invasive species: The effects of time since invasion and population management history on beavers in Tierra del Fuego, Argentina. J. Zool..

[40] FAO (GLOBAL ENVIRONMENT FACILITY). *Strengthening and Development of Instruments for the Management, Prevention and Control of Beaver (Castor canadensis), an Invasive Alien Species in the Chilean Patagonia* 125. https://www.thegef.org/projects-operations/projects/5506 (2016).

[41] Pietrek AG, González-Roglich M (2015). Post-establishment changes in habitat selection by an invasive species: Beavers in the Patagonian steppe. Biol. Invas..

[42] Collado L (2001). Los bosques de Tierra del Fuego. Análisis de su estratificación mediante imágenes satelitales para el inventario forestal de la provincia. Multequina.

[43] Morra B, Brisbin H, Stringham T, Sullivan BW (2023). Ecosystem carbon and nitrogen gains following 27 years of grazing management in a semiarid alluvial valley. J. Environ. Manag..

[44] Westbrook CJ, Cooper DJ, Anderson CB (2017). Alteration of hydrogeomorphic processes by invasive beavers in southern South America. Sci. Total Environ..

[45] Baldini UA, Oltremari AJ, Ramírez M (2008). Impacto del castor (*Castor canadensis*, Rodentia) en bosques de lenga (*Nothofagus pumilio*) de Tierra del Fuego, Chile. Bosque Valdivia.

[46] Wallem PK, Jones CG, Marquet PA, Jaksic FM (2007). Identificación de los mecanismos subyacentes a la invasión de *Castor canadensis* (Rodentia) en el archipiélago de Tierra del Fuego, Chile. Rev. Chil. Hist. Nat..

[47] Gorelick N (2017). Google earth engine: Planetary-scale geospatial analysis for everyone. Remote Sens. Environ..

[48] Alencar A (2020). Mapping three decades of changes in the Brazilian Savanna native vegetation using landsat data processed in the google earth engine platform. Remote Sens..

[49] Rebertus AJ, Veblen TT (1993). Structure and tree-fall gap dynamics of old-growth *Nothofagus* forests in Tierra del Fuego, Argentina. J. Veg. Sci..

[50] Corporación Nacional Forestal (CONAF). *Incendios Forestales en Chile: Estadísticas históricas*. https://www.conaf.cl/incendios-forestales/incendios-forestales-en-chile/estadisticas-historicas/ (2023).

[51] Bey A (2016). Collect earth: Land use and land cover assessment through augmented visual interpretation. Remote Sens..

[52] Elith J, Leathwick JR, Hastie T (2008). A working guide to boosted regression trees. J. Anim. Ecol..

[53] Hijmans, R. J., Phillips, S., Leathwick, J. R. & Elith, J. *Species Distribution Modelling Package ‘dismo’* 68 (2021).

[54] Barrera MD, Frangi JL, Richter LL, Perdomo MH, Pinedo LB (2000). Structural and functional changes in *Nothofagus pumilio* forests along an altitudinal gradient in Tierra del Fuego, Argentina. J. Veg. Sci..

