# Supplementary material for: Equivalent impacts of logging and beaver activities on aboveground carbon stock loss in the southernmost forest on Earth
Source: Sci Rep. 2023 Oct 26;13:18350. doi: 10.1038/s41598-023-45657-4 (PMC10603114; doi:10.1038/s41598-023-45657-4)
Supplement: Supplementary file 1 — Supplementary Information 1. [file 41598_2023_45657_MOESM1_ESM.docx]

**Supplementary material 1**

**Equivalent impacts of logging and beaver activities on aboveground carbon stock loss in the southernmost forest on Earth**

Alejandro Miranda, Jorge Hoyos-Santillan, Antonio Lara, Rayén Mentler, Alejandro Huertas-Herrera, Mónica D. R. Toro-Manríquez, Armando Sepulveda-Jauregui

| **Supplementary material 1**. List of predictor variables initially used for classification of forest loss drivers. | | | |
| --- | --- | --- | --- |
| **Climate:** | **Source** | **Process** | **Resolution** |
| 1. Annual precipitation (mm)* | CR2met** | Mean value. | 5 Km |
| 2. Mean maximum winter temperature (°C)* | CR2met** / developed in this research | From the monthly temperature raster, a maximum temperature raster was obtained for the winter months of each year, then the average value for the whole period was calculated. | 5 Km |
| 3. Mean minimum winter temperature (°C)* | CR2met** / developed in this research | From the monthly temperature raster, a minimum temperature raster was obtained for the winter months of each year, then the average value for the whole period was calculated. | 5 Km |
| 4. Annual evapotranspiration | https://cgiarcsi.community/ |  | 1 Km |
| 5. Mean minimum summer temperature (°C), | CR2met** / developed in this research | From the monthly temperature raster, a minimum temperature raster was obtained for the summer months of each year, then the average value for the whole period was calculated. | 5 Km |
| 6. Mean maximum summer temperature (°C) | CR2met** / developed in this research | From the monthly temperature raster, a maximum temperature raster was obtained for the summer months of each year, then the average value for the whole period was calculated. | 5 Km |
|  | | | |
| **Topography and geography:** | | | |
| 7. Latitude (°)* | Developed in this research | Calculated with ArcGis, as the midpoint of the grid with EPSG: 4326 WGS 84. |  |
| 8. Longitude (°)* | Developed in this research | Calculated with ArcGis, as the midpoint of the grid with EPSG: 4326 WGS 84. |  |
| 9. Elevation (m a.s.l.) | CGIAR/SRTM90_V4 product  https://earthengine.google.com/ |  | 90 m |
| 10. Terrain slope (°) | https://earthengine.google.com/ | Calculated in GEE from Elevation product. | 90 m |
| 11. Topographic Wetness Index | Developed in this research | Calculated in QGIS SAGA from slope product. | 30 m |
|  | | | |
| **Indicators of human activity and beaver’s presence:** | | | |
| 12. Dams’ density (dams/km^2^)* | <http://dx.plos.org/10.1371/journal.pone.0232057> |  | 30 m |
| 13. Distance to the nearest road (km)* | Developed in this research | Calculated in ArcGis with euclidean distance. | 30 m |
| 14. Distance to the nearest town (km) | Developed in this research | Calculated in ArcGis with euclidean distance. | 30 m |
| 15. Distance to the nearest watercourse (km) | Developed in this research | Calculated in ArcGis with euclidean distance. | 30 m |
|  | | | |
| **Land cover characteristics relevant for logging and beavers:** | | | |
| 16. Shrub cover in 2019 (%)* | Developed in this research | Percentage calculated based on a landcover created with supervised classification using Landsat images for the year 2019. | 30 m |
| 17. Shrub cover in 1986 (%) | Developed in this research | Percentage calculated based on a landcover created with supervised classification using Landsat images for the year 1986. | 30 m |
| 18. Forest cover in 1986 (%) | Developed in this research | Percentage calculated based on a landcover created with supervised classification using Landsat images for the year 1986. | 30 m |
| 19. Net primary production | <http://files.ntsg.umt.edu/> |  | 1 Km |
| 20. Peatland cover in 2019 (%) | Developed in this research | Percentage calculated based on a landcover created with supervised classification using Landsat images for the year 2019. | 30 m |
| 21. Peatland cover in 1986 (%) | Developed in this research | Percentage calculated based on a landcover created with supervised classification using Landsat images for the year 1986. | 30 m |
| 22. Bare land cover in 2019 (%) | Developed in this research | Percentage calculated based on a landcover created with supervised classification using Landsat images for the year 2019. | 30 m |
| 23. Bare land cover in 1986 (%) | Developed in this research | Percentage calculated based on a landcover created with supervised classification using Landsat images for the year 1986. | 30 m |
| 24. Water cover (%) | Developed in this research | Percentage calculated based on a landcover created with supervised classification using Landsat images for the year 2019. | 30 m |
| 25. Forest loss (%)* | Developed in this research | Percentage calculated based on the difference between the landcovers of 2019 and 1986. | 30 m |
| 26. Area of adjacent grids with forest loss (km^2^) | Developed in this research | Calculated in QGISl |  |
| *Indicates variables used in the final model.  **CR2met (https://www.cr2.cl/datos-productos-grillados/) | | | |
