# Supplementary material for: Equivalent impacts of logging and beaver activities on aboveground carbon stock loss in the southernmost forest on Earth
Source: Sci Rep. 2023 Oct 26;13:18350. doi: 10.1038/s41598-023-45657-4 (PMC10603114; doi:10.1038/s41598-023-45657-4)
Supplement: Supplementary file 2 — Supplementary Information 2. [file 41598_2023_45657_MOESM2_ESM.docx]

**Supplementary material 2**

**Equivalent impacts of logging and beaver activities on aboveground carbon stock loss in the southernmost forest on Earth**

Alejandro Miranda, Jorge Hoyos-Santillan, Antonio Lara, Rayén Mentler, Alejandro Huertas-Herrera, Mónica D. R. Toro-Manríquez, Armando Sepulveda-Jauregui

**2.1.**

| **Aboveground biomass (AGB) of *N. pumilio* forests in Karukinka, Tierra del Fuego, Chile; adapted from Harris *et al.* 2008** | | | |  |  |
| --- | --- | --- | --- | --- | --- |
| Carbon pool | Undisturbed *N. pumilio* forest (tCO_2_ ha^-1^)^a^ | Beaver impacted (tCO_2_ ha^-1^)^b^ | Logging impacted (tCO_2_ ha^-1^)^c^ | |  |
| Trees (> 5cm diameter) | 488 | 110 | 272 | | |
| Standing dead wood | 41 | 162 | na | | |
| Lying dead wood | 171 | 242 | 171 | | |
| Total | 700 | 514 | 443 | | |
| ^a^AGB data for Undisturbed *N. pumilio* plots from Table 2 and 14 Harris et al. 2008 (10 plots; SD = 186; 95 % Confidence = 131) | | | | |  |
| ^b^AGB data for Beaver disturbed *N. pumilio* plots from Table 2 and 14 Harris et al. 2008 (9 plots; SD = 309; 95 % Confidence = 233) | | | | |  |
| ^c^AGB data for Logging disturbed *N. pumilio* plots from Table 2 Harris et al. 2008 (9 plots; SD = 63; 95 % Confidence = 63) | | | |  |  |

**2.2. Contribution of logging and beavers’ activity to carbon loss between 1986 and 2019.**

$Carbon stock loss \left( tC \right)= \sum_{t=1}^{33} \sum_{i=1}^{4} \left( ({IAGB}_{l} +D{AGSDB}_{l}+D{AGLDB}_{l} \right)+\left( {IAGB}_{b} +D{AGSDB}_{b}+D{AGLDB}_{b} \right))$ (1)

Where: *t* = time between 1986 and 2019 (33 years); *i* = altitude gradient (4 altitude ranges); IAGB_l_ = initial AGB carbon loss due to logging; DAGSDB_l_ = decay aboveground standing dead biomass due to logging; DAGLDB_l_ = decay aboveground lying dead biomass due to logging; IAGB_b_ = initial AGB carbon loss due to beavers’ activity; DAGSDB_b_ = decay aboveground standing dead biomass due to beavers’ activity; DAGLDB_b_ = decay aboveground lying dead biomass due to beavers’ activity.

**2.3.**

| **Exponential decay models for *Nothofagus pumilio* for wood in Tierra del Fuego^a^** | | | |
| --- | --- | --- | --- |
| *N. pumilio* wood | Equation | r^2^ | P |
| Branches (8-17 cm Ø) | $y=76.31 \cdot e^{-0.019 \cdot t}$ | 0.63 | < 0.001 |
| Logs (19-60 cm Ø) | $y=83.70 \cdot e^{-0.010 \cdot t}$ | 0.76 | < 0.001 |
| ^a^Models developed by J. L. Frangi, L. L. Richter, M. D. Barrera, M. Aloggia, Decomposition of Nothofagus fallen woody debris in forests of Tierra del Fuego, Argentina. *Can. J. For. Res.* **27**, 1095–1102 (1997). | | | |
